# Impact of the participation of rural households in the appraisal on poor households’ identification

**DOI:** 10.1371/journal.pone.0272519

**Published:** 2022-08-05

**Authors:** Yusong Liu, Linyi Zheng

**Affiliations:** 1 China Academy for Rural Development, Zhejiang University, Hangzhou, Zhejiang, China; 2 School of Public Administration, Hunan University, Changsha, China; Shenzhen University, CHINA

## Abstract

Developing countries have common goals of poverty eradication and improving people’s livelihoods. As the largest developing country, China has made remarkable achievements in poverty alleviation during the 30 years of reform. Although a targeted poverty alleviation mechanism was established by the Chinese government in 2013, the identification of poor households has been an arduous journey. Based on a total of 688 samples of grassroots officials and 2,621 rural households from 69 village-level divisions in 9 provinces in China, this study conducted cross-validation on the impact of the participation of rural households in the identifying poor households that required government assistance. This was from the perspectives of grassroots officials and rural households. It was investigated whether this participation led to an anomaly between the identification of poor households and the actual situation. Empirical results show that the participation of rural households in appraisals significantly increases the probability of identifying a non-poor household as a poor household (first error) and decreases the probability of failing to identify a poor household as a poor household (second error). As the impact of the first error is greater than that of the second error, the participation of rural households in appraisals has the overall effect of increasing the incorrect registrations of poor households. These results are still valid after addressing the self-selection problem. For other developing countries to successfully apply effort into poverty alleviation, in addition to focusing on increasing farmers’ participation in public affairs, they should prevent any bias that may be caused by farmers’ participation in public affairs; strengthen publicity and guidance; focus on the nurture of officials; perfect top-level design; and set clearer targets for poverty alleviation policies.

## Introduction

As of 2020, according to the rural poverty standard of 2,300 RMB (based on constant 2010 prices) per person per year, all 98.99 million rural poor people have been lifted out of poverty, accomplishing the historical feat of eradicating absolute poverty in China. According to the poverty line of 1.9 USD used by the World Bank, the poverty incidence of China decreased from 6.03% in 2011 to 0.2% in mid-2019. The poor population had therefore decreased by over 80 million, and the decline was the largest in the world [1]. When we examined the data of other countries from 2011–2019, we ascertained that the poverty incidence of Brazil had increased from 3.42% to 4.24%, while the poor population had increased by 2.1969 million; the poverty incidence of South Africa had decreased from 25.09% to 24.45% but the poor population had increased by 1.2453 million; the poverty incidence of India had decreased from 12.61% to 7.41% and the poor population had decreased by 57.3566 million. With almost 50 years of unremitting efforts, China’s poverty alleviation, which is led by administrative work, has achieved brilliant successes along with worldwide recognition. What cannot be ignored, however, is that problems have arisen in the process of China’s poverty alleviation, including unclear numbers and statuses of poor residents, weak targeting, and inaccurate directions of poverty alleviation funds and projects, which deserve attention from other developing countries [2].

The precise identification of poor households is the prerequisite and foundation for the implementation of targeted poverty alleviation. If the results of poor household identification (i.e., poverty registration) are inaccurate, targeted poverty alleviation is not possible. Under the constraints of poverty alleviation resources, poverty identification is a worldwide problem. Coady et al. [3] conducted a study on 122 poverty alleviation intervention policies in 48 countries and found that the poor were not effectively identified in 25% of the projects. Similarly, the research of Platteau [4] on West Africa’s social fund project, Galasso et al. [5] on Bangladesh’s food education program and Pan and Christiaensen [6] on Tanzania’s subsidy project on agricultural input highlighted that there were deviation problems in the identification of poverty alleviation efforts. Similar problems have arisen in China’s targeted poverty alleviation. Deviation problems with identification have occurred in the registration of poverty, which is part of the process of targeted poverty alleviation and identifies the household as a unit [7–11]. A set of data from the State Council Leading Group Office of Poverty Alleviation and Development shows that from August 2015 to June 2016, nearly two million people across the country were mobilized to conduct “Follow-Up Checks” on poverty registration, in which the poor households that had already been precisely identified were investigated. Exactly 9.29 million people who had been wrongly identified were removed from the registration, while 8.07 million newly identified people were added [12].

Why are there still omissions and mismatches in China’s precise identification? Some scholars believe that as the poverty alleviation resources are mostly controlled by officials, the phenomenon of “rent-seeking” may occur due to the corruption of grassroots officials, while there may also be “deviations and change of poverty alleviation targets” due to the decisions of rational people [13, 14]. Meanwhile, non-poor households will compete for poverty alleviation resources [15]. Some scholars also highlighted that many poor or non-poor households had not actively participated in poverty registration and had given little support to the registration effort as they believed that it had nothing to do with them [16]. The resulting bias will negatively impact government efforts. Although the abovementioned studies have provided reasonable explanations and laid the foundation for the in-depth research of this study, few studies have investigated the impact of the participation of rural households in the appraisal. This includes the identification of poor households from the perspective of the implementation process. Studies have shown that apart from affecting the satisfaction that rural households have with public policies, participation is also a factor with a decisive impact on the implementation effect of public policies [17]. Its importance in the process of targeted poverty alleviation cannot be underestimated. In fact, our survey data also illustrates this point. According to statistics, 42.19% of rural households highlighted that the process of identifying poor households was not objective or fair. Furthermore, considering that government failure is primarily reflected in equal participation in public policies and the openness and transparency of public policies [8], in this study, this failure was evaluated by examining whether rural households can participate in identifying poor households. Accordingly, the impact of their participation in this appraisal was examined. It is hoped that the results can provide reference for the decision-making of developing countries in poverty alleviation, while providing helpful suggestions for China to build an open, transparent, fair, and honest service-oriented government.

### Institutional background, literature review and theoretical analysis

#### Institutional background

On April 2, 2014, in line with the spirit of “The Notice of ‘Opinions on Innovating Mechanism to Promote Rural Poverty Alleviation and Development’ by the Central Committee” (No. 25 [2013] of the general office of the CPC Central Committee), the State Council Leading Group Office of Poverty Alleviation and Development formulated “The Plan for Poverty Alleviation Registration” according to the working requirements for poverty registration determined by the National Conference on Development-driven Poverty Alleviation and the second meeting of the State Council Leading Group of Poverty Alleviation and Development:

1. Work goals

The targets of poverty registration include poor households, poor villages, poor counties, and contiguous poverty-stricken areas. Through the process of poverty registration, the authorities are required to conduct the precise identification of poor households and poor villages, obtain a general picture of their situations, the causes of their poverty, and their particular needs for poverty elimination in order to provide concrete help, implement assistance measures, carry out assessments and inquiries on the efficacy, and implement dynamic management. They also have to monitor and evaluate the situations of poor counties and contiguous poverty-stricken areas, analyze and grasp the situation of poverty alleviation and development, and provide a basis for the decision-making and assessment in poverty alleviation and development. The authorities were required to establish electronic information files that include the information of poor households, poor villages, poor counties and contiguous poverty-stricken areas across the country, and distribute the “Poverty Alleviation Handbooks” to poor households by the end of 2014. On this basis, they were required to build a national poverty alleviation information network system to lay the foundation for targeted poverty alleviation.

2. Methods and steps for poverty registration
(1) Working methods
  (a) Standards. The national rural poverty standard of 2,736 RMB (equivalent to the constant price in 2010, i.e., 2,300 RMB) per capita net income of farmers in 2013 is taken as the identification standard. When a province, autonomous region, or a municipality (hereinafter collectively referred to as provinces) has ensured the completion of the identification task according to the national rural poverty standard, the province begins the subsequent work of poor household identification according to its own situations and standards, and registers the relevant data in the national poverty alleviation information network system for unified management. (b) Size. In principle, the rural poor population size of 82.49 million at the end of 2013, which was disclosed by the National Bureau of Statistics, is taken as the base. (c) Methods. Scale control was adopted. The scale of poor people identification of each province is divided level-by-level, down to village-level divisions. The identification of poor households should be based on the income of farmers. Other criteria include housing, education, and health. Each rural household is treated as one unit and is required to go through the steps of applications, democratic appraisals, public announcements, and level-by-level review, before it is identified as a poor household. (d) Contents of registration. The *Poverty Alleviation Handbook* of each household records the basic family conditions, causes of poverty, the contact official in charge of poverty elimination, the poverty alleviation plan, and the efficacy of poverty alleviation. The standard time for registration is December 31, 2013, and the standard period is from January 1, 2013 to December 31, 2013.
(2) Work steps and schedule

**Fig 1:**
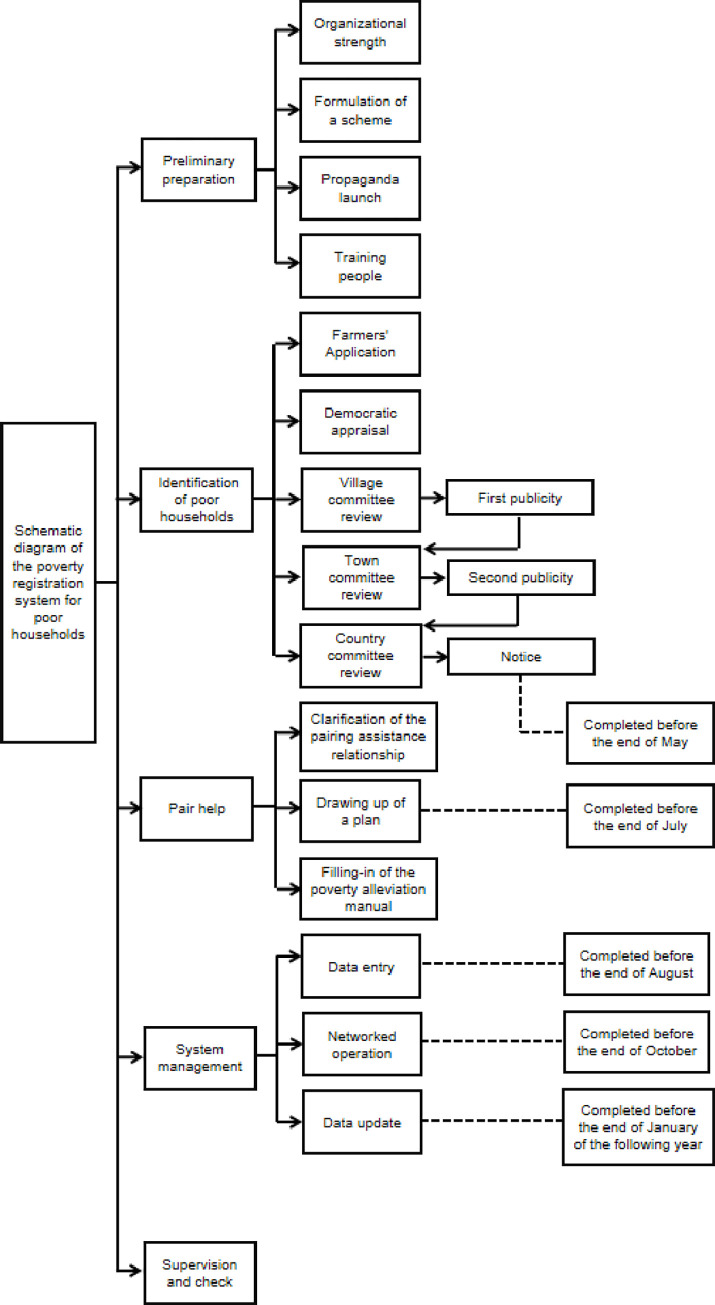
Functional diagram of the poverty registration of poor households.

The working process and reference text for the poverty registration of poor households are shown in Fig 1.

## Literature review

Existing studies on targeted poverty alleviation at home and abroad mainly focus on the connotations of targeted poverty alleviation, the implementation results, the difficulties and the ways of realizing targeted poverty alleviation.

Regarding the connotation and definition of targeted poverty alleviation, Wang and Guo [18] pointed out that the most basic definition of targeted poverty alleviation is that the policies and measures of poverty alleviation should target the families and population that are truly poor, remove the root causes and obstacles that lead to poverty with well-targeted help for the poor population, and achieve the goal of sustainable poverty alleviation. Zheng and Wang [19] believe that targeted poverty alleviation originated from the adjustment of poverty alleviation ideas in Western welfare societies during periods of tight finances. The essence of this adjustment is consistent with that of China’s poverty alleviation and development policies, which has transformed from a universal-type to a moderate-competitive-type of social welfare. The focuses are on particularly targeting the wills of the poor, streamlining the system in which poverty alleviation resources assert the effects, as well as reconstructing the functional process, in order to improve the efficiency of poverty alleviation, and promote the process of lifting the poor out of poverty.

Regarding the implementation results of targeted poverty alleviation, Zhu and Mo [2] pointed out that the identification model with officials spending time in villages has ensured the precise identification of the poor groups; the assistance model that involves the participation of multiple entities has promoted the allocation of social poverty alleviation resources; while the new assessment method of poverty alleviation efficacy has improved the efficiency of poverty alleviation and development. Based on first-hand data retrieved from door-to-door household surveys on targeted poverty alleviation conducted in Shaanxi Province, Cao et al. [17] investigated farmers’ satisfaction with targeted poverty alleviation policies and its influencing factors from five dimensions, namely the rural households’ location, individual characteristics, family income characteristics, poverty statuses and their correlation with poverty alleviation policies. Using the PSM method and taking Pengyang County of Ningxia as an example, Shi and Wen [20] conducted a study on the multidimensional poverty reduction effect of targeted poverty alleviation from the angle of comparative poverty, and the results showed that targeted poverty alleviation resulted in significant multidimensional poverty reduction effects. The study of Elbers et al. [21] showed that when recently completed “poverty maps” for three countries were employed as the tools to transfer an exogenously given budget to geographically defined poor areas or villages according to the population’s relative poverty status, huge poverty alleviation benefits would be obtained. The research results of Tsai et al. [22] demonstrated that targeted poverty alleviation policies for the poor could effectively reduce the AIDS cases in Africa.

Regarding the difficulties arising in the process of targeted poverty alleviation, Wang and Xing [23] identified that China’s work mechanism of targeted poverty alleviation is hindered by problems such as the inaccurate identification of service targets, flawed mechanism of dispatching poverty alleviation officials to villages, insufficient endogenous motivation for industrialization-based poverty alleviation, difficulties in integrating poverty alleviation funds and unbalanced allocation of poverty alleviation resources. Shen [24] believes that targeted poverty alleviation policies are prone to be restricted by the dimensions of ecology, nature, socio-economy, demographic, institution and policy, as well as poverty alleviation costs. Sun and Sun [25] maintained that in the implementation of targeted poverty alleviation policies, the phenomenon of “seas of forms” is prone to appear. A significant number of forms have to be completed and severely reduces the efficiency of grassroots officials.

Regarding the methods of realizing targeted poverty alleviation, Deng [7] believes that to ensure the realization of the goal of targeted poverty alleviation, it is necessary to ensure precise identification, assistance, and scientific dynamic management. Gong [13] believes that the market-based approach to poverty alleviation has advantages in terms of accuracy and efficiency. China’s market mechanism has been relatively mature and complete, while market power has been widely used in the practices of poverty alleviation in various regions. In future, the proportion of market-based approaches can definitely be increased in the practices of poverty alleviation. Liu [26] suggested that the slowdown of market reforms had significantly aggravated rural poverty. The study of Kydd and Dorward [27] showed that the establishment and development of free markets had not brought about a widespread growth of agriculture, while rural poverty had not been significantly reduced. However, the studies of Khan and Riskin [28] and Sicular et al. [29] maintained that market-oriented reforms had reduced poverty in rural China.

Examining the literature, the following phenomena are obvious: First, there are more normative studies on precise identification, while there are less quantitative analyses. The use of quantitative analysis in the study on precise identification is more scientific, reasonable, and convincing. Second, existing quantitative analyses are confined to one region in each study, while there have not been any large sample surveys conducted across the entire country. In the field of economic research, this may lead to sample selection bias. Third, as the assessments of satisfaction were conducted based on the overall policies, the results were too general and the satisfaction with specific parts were not revealed, therefore the problems could not be pinpointed. Consequently, it is meaningful to collect survey data on-site and analyze poor household identification under the precise identification mechanism with econometric methods. On the one hand, this research can help discover the causes of government failures and provide a reference for the government’s administration; on the other hand, this research can also urge grassroots officials to correct the deviations as soon as possible and set clearer targets for targeted poverty alleviation.

### Theoretical analysis and research hypotheses

Regarding the connotation of service-oriented government, the new public service theory proposed by American scholar Denhardt [30] holds that the role of government is to serve instead of to steer, and that public interest is a goal rather than a by-product. The goal of China’s targeted poverty alleviation policies is to realize the precise identification of actual poor households and use the poverty alleviation resources with clear targets, thus ultimately to achieve common prosperity for everyone. Grassroots-level poverty alleviation work, including the promotion of targeted poverty alleviation policies, the training of poverty alleviation officials, the indicators of poor household identification, and the publication of the poor household identification process, all of which are parts of the precise identification mechanism, will involve public information services that are an essential part of government services. According to the basic framework of information economics, entities active in the market will definitely collect effective information by all means and make decisions based on the information they collect [31]. Feng [32] believes that public information services is the process in which the information related to public interests, public policy formulation, arrangements, and implementation of the public management system, as well as public affairs management activities, are opened and developed for the services.

At the same time, the core of a service-oriented government lies in fairness and efficiency. The government realizes public management by providing public services to the public. Zhang [33] showed that there are government failures in grass-roots public information services. This phenomenon is mainly reflected in the emergence of information vulnerable groups, which refer to the social groups and individuals who are at a disadvantage in obtaining information and using information infrastructure in an information society. In targeted poverty alleviation work, information vulnerable groups refer to the poor households that are unable to obtain information on poverty alleviation policies. Due to their inability to access public information and information asymmetry, information vulnerable groups are at a disadvantage in accessing public services. Although numerous operating mechanisms and evaluation indicators have to be formulated for targeted poverty alleviation to reduce the inequity in poor household identification, it is not easy to judge the actual effect from appearances. The key to minimizing these particular government failures is to discover if equity problems exist within the precise identification mechanism. Government service satisfaction surveys are one of the solutions. However, it is difficult to observe whether there is any inequity in public information services from satisfaction alone. Therefore, a reasonable data analysis model is required to discover these failures in services. We believed that an effective democratic method was to examine the participation of rural households in the appraisal process. We surmised that farmers’ identification of poor households would have a significant effect on the bias or otherwise of the appraisal.

In response to the question “Do you believe that the current poor household identification is objective and fair?” only 57.23% of rural households thought that the process for identifying poor households was objective and fair. Meanwhile, 569 grassroots officials believed that the current poor household identification was objective and fair, accounting for 82.70%, but nearly one-fifth of the officials had opposite opinions. The main reasons discovered in the survey are as follows: “Some family members are always out of the villages and cannot participate in the poor household identification,” “The information of poor household identification is monopolized and the participation in poverty identification is impossible,” “It is easy to be affected by people’s relations with government officials (“Guanxi hu,” people who have connections with officials),” and “The panel is unfair in its decisions.” The fair distribution of government poverty alleviation resources involves three considerations of equity: opportunity equity, procedure equity, and result equity [15]. Several issues should be considered in poor household identification: Who will make the assessment, how it will be assessed, who will supervise the assessment, whether the decision has been publicized, how it will be publicized, where it will be publicized, and whether the public have objections. Under normal circumstances, villagers participate in the democratic appraisal of poor household identification, and the list of poor households is publicized after the assessment. Whether the assessment process of local poor households is open and transparent will have a significant impact on the results of the evaluation [34]. Democratic evaluation is an important link between village public decision-making and resource allocation. The common practice is to vote first and then verify according to village cadres. Theoretically speaking, such a mechanism can function to realize the equitable distribution of poverty alleviation resources. However, practice has indicated that the actual effects of the mechanism are not like this, and the reasons are as follows: First, elite lobbying occurs, via which village leaders or people with power may lobby the villagers to solicit votes, and village cadres and other insiders may also intercept or inflict bias; Second, the self-interested motives and egalitarian concepts of ordinary villagers, whereby the notion of personal gain will affect the voting behavior of ordinary villagers; Third, administrative control, which can be explained as follows: in order to achieve the goal of poverty alleviation more smoothly, the grass-roots government selects villagers who fulfill relevant criteria [35]. Grassroots governments and village “two committees” tend to make effective use of their information advantage relative to their superiors to identify and report "poor households" that meet the interests of their own level, but are not necessarily poor, and successfully achieve the goal of skewing the identification of poor households [8]. Although poor household identification is a relatively important matter, in the area where the respondents were located, a large number of villagers were not able to join the democratic appraisals (78 cases), and the results after the appraisals were not publicized in many cases (43 cases). For this reason, this paper proposes Hypothesis 1, which verifies whether the participation of rural households in the appraisal leads to deviation in the identification results of poor households.

Hypothesis 1: The participation of rural households in the appraisal will reduce the probability of wrongly identifying poor households.

Due to the flaws in the implementation of the democratic appraisal system, up to one-third of the officials believed that some poor families had been omitted while some resources had been wrongly allocated to non-poor families during the implementation of the current poverty alleviation policies. The main reason for the exclusions in the identification process was that poor families had little understanding of the poverty alleviation projects. Other reasons were that non-poor families obtained the resources through various illegitimate connections, poor families were unable to join the process, the government had rigid arrangements, or that poor families were unwilling to participate. Inadequate publicity by the government opens loopholes for some people to exploit the system [7, 25]. Exactly 37.50% of the officials believed that the rural people in their areas were fighting fiercely for the “badge of poverty.” Furthermore, 34.48% of the officials even believed that there were cases in which farmers concealed their real income information (such as misstatement of income and hiding of assets). Unfairness in the appraisals can easily lead to mass incidents. As many as 210 officials pointed out that farmers who had not been classified as poor households would lodge petitions with the authorities. Several studies have also confirmed this. Non-poor groups can often take advantage of their economic position to obtain welfare service policies that are beneficial to themselves [36]. Village administrative heads are more likely to obtain quantitative welfare cards issued to the poor [37]. Elite capture exists in food education programs in Bangladesh [5]. In Tanzania, there is an obvious phenomenon of elite capture in the issuance of coupons for agricultural input subsidy projects. The families of village cadres have obtained 60% of coupons for agricultural input subsidy projects [6]. There is a widespread phenomenon of elite capture in the beneficiary identification of public welfare projects in low-income countries, and the beneficiary identification and process of projects are seriously affected by the elite capture of grassroots local governments [38]. Elite lobbying exists at the stage of democratic evaluation in China, that is, relatively rich rural classes use their network in villages to lobby or solicit votes by giving money and goods in the process of democratic evaluation, aiming to achieve the substantial results of controlling poverty alleviation projects through democratic forms and procedures [35]. In a study conducted by Hu and Wang [9], it is found that many villagers’ representatives are not elected by the village group but appointed by the village head and party secretary, and the convening of the villagers’ congress depends on the personal wishes of the village head and party secretary. As a result, some deeply poor peasant households cannot become registered households, while some elite peasant households who have good relations with the village head and party secretary become registered households. Due to a lack of information, cadre corruption, elite capture, and other factors, compared with elite farmers, poor households participate in the evaluation to a lower degree and have a lower probability of obtaining a registration card [15, 16]. According to the survey, the elite capture rate of registration cards is 25%, that is, 25 households out of 100 poverty-registered households are occupied by elite farmers. The registration card system faces severe challenge from this elite capture [9]. Identification procedures that involve villagers effectively should be adopted. Therefore, based on the statistics of “discarding the real” and “remaining the wrong” the principle of two types of errors, we further proposed Hypothesis 2 and Hypothesis 3 to verify whether the participation of rural households in the appraisal would change the probability of non-poor households being identified as poor households and the actually poor households not being identified as poor households.

Hypothesis 2: The participation of non-poor households in the appraisals may increase their probability in being identified as poor households.

Hypothesis 3: If the poor households do not participate in the appraisals, their probability of being identified as poor households will decrease.

## Research design

### Data source

The data used in this study comes from a key project of the National Social Science Fund of China, “Diversified Path Design and Exit Mechanism for Targeted Poverty Alleviation in Poverty Regions.” In 2016, the project research team conducted field surveys in nine provinces, municipalities and autonomous regions including Shandong, Shanxi, Sichuan, Gansu, Yunnan, Guizhou, Hunan, Chongqing and Tibet. Stratified random sampling design was used in the survey while the provinces chosen were those with larger poor populations. The questionnaire was divided into two parts: (1) Questionnaire for grassroots officials. This section involved the systematic investigation of the rural grassroots officials’ perception of targeted poverty alleviation, the evaluation of the government’s diversified poverty alleviation policies, and the exit mechanism of poor households. Respondents were required to have rural household registration statuses and to have served as grassroots officials in rural areas. In this section, 796 questionnaires were collected, among which 688 were valid, with an effective rate of 86.43%. (2) Questionnaire for farmers. This section involved the systematic investigation of the basic conditions of farmers and the situations in targeted poverty alleviation for poor households. Respondents were required to have rural household registration statuses, while only one member was selected for the survey in each household. In this section, 2,661 questionnaires were collected, among which 2,621 were valid, with an effective rate of 98.50%. The data were collected from the different perspectives of grassroots officials and farmers so that the research could be cross-validated.

### Variable selection

Through the previous analysis and literature review, we found that the reasons for the poverty of poor households are very complicated [16, 17, 39–41]. Accordingly, with reference to previous studies, we selected indicators from various aspects to study the factors affecting the precise identification of poor households. The definitions and assigned values of the variables are shown in Table 1.

**Table 1:** Summary of the variables.

Variable		Value	Mean	Standard deviation	Min	Max
Dependent variable	Y1: Identifying a non-poor household as a poor household	Yes = 1; No = 0	0.192	0.394	0	1
	Y2: Failure to identify a poor household as a poor household	Yes = 1; No = 0	0.123	0.329	0	1
	Y3: One of the above two situations occurs	Yes = 1, No = 0	0.315	0.465	0	1
Core independent variable	Participation in appraisals	Yes = 1; No = 0	0.648	0.478	0	1
	Government failure	Yes = 1; No = 0	0.572	0.495	0	1
Control variables	Gender	Male = 1; Female = 0	0.812	0.391	0	1
	Age	Age of head of household	48.944	12.615	16	73
	Marital status	Married = 1; Without a spouse = 0	0.839	0.368	0	1
	Educational level	Illiterate = 1; Elementary school = 2; Junior high school = 3; High school = 4; College and above = 5	2.182	0.879	1	5
	Understanding of policies	Degree of understanding the policies: Completely do not understand = 1; Do not understand much = 2; Understand a little = 3; Relatively better grasp = 4; Fully understand = 5	2.456	0.917	1	5
	Family size	Total number of family members	4.26	1.642	1	13
	Labor size	Number of members that can work	2.295	1.061	0	6
	Non-agricultural employment	Number of members engaged in non-agricultural industries	1.016	0.984	0	6
	Land size	Area of arable land	4.002	5.653	0	240
	Houses value	Value of house price, logarithmically transformed value	10.875	1.184	0	14.286
	Family income	Household income, logarithmically transformed value	10.075	1.133	0	13.605
	Family expenditure	Household expenditure, logarithmically transformed value	9.863	0.893	6.399	13.456
	Plains	Yes = 1; No = 0	0.104	0.306	0	1
	Hills	Yes = 1; No = 0	0.039	0.193	0	1
	Mountains	Yes = 1; No = 0	0.857	0.35	0	1

1. Dependent variable. The dependent variable in this study is the precise identification of poor households. The question, “Is your family recognized as a poor household? (that is, filed in poverty registration)” was selected as the identification criterion of poor households, while three dummy variables were also included in the model: (1) Identifying a non-poor household as a poor household (first type of error); (2) failure to identify a poor household as a poor household (second type of error); (3) One of the above two situations occurs.
2. Core independent variable. Based on the aforementioned research, the question, “Can villagers participate in the democratic appraisal of poor household identification in your area?” was selected to measure the implementation of targeted poverty alleviation policies. Concurrently, the question, “Do you think the identification of poor households is objective and fair?” was selected for a robustness test.

Control variables. To make the model more scientific and reasonable, it is usually necessary to include some control variables. With reference to Gustafsson and Wei [42], Wang and Sabina [43], Cao et al. [17], Cheng et al. [39], Wang and Yin [44] and Du et al. [45], the following control variables were chosen in this study: (1) The situation of the head of the household. As the head of a family, the head of the household is usually the main breadwinner of the family. Whether the head of the household is young, energetic, and well-educated are the crucial factors in whether an individual family can ameliorate poverty. Therefore, the gender, age, marital status, and educational level of the head of the household were selected as indicators of the situation. (2) Family situation. The physical capital and human capital of each rural family are different. Therefore, the area of the arable land and the value of houses owned by a family were selected as indicators of physical capital, while the total number of family members (includes members of all ages), the number of members that can work, and the number of members engaged in non-agricultural industries were selected as the indicators of human capital. (3) Family income and expenditure. The annual income and expenditure of rural households also affects poor household identification. Therefore, the total income and total expenditure of a rural household in the previous year were selected as indicators to measure the financial conditions of a rural household. Among them, family income was taken to include but was not limited to income from agricultural production, income from non-agricultural industries, transfer income (government subsidies), and property income (leased land, equipment, etc.). Expenditure included but was not limited to food consumption, clothing consumption, housing construction and renovation expenditure, household appliance expenditure, transportation and communication expenditure, culture and entertainment expenditure, medical care expenditure, and gift expenditure. (4) Other factors. Farmers’ understanding of the targeted poverty alleviation policies and the terrain of the area where the farmers are located were used to measure the effects of other factors. The topographic features were treated in the form of dummy variables.

### Descriptive statistics

#### Analysis of the poverty registration of poor households

With reference to the national standard line in 2015, rural households with a per capita net income (per capita net income = family income/family size) of less than or equal to 2,800 RMB in the previous year were divided into two groups: poor households (557 samples in total) and non-poor households (2,064 samples in total). Simultaneously, according to the poverty identification results, the samples were divided into two groups: rural households filed in the poverty registration (737 samples in total) and rural households not filed in the poverty registration (1,884 samples in total). Only 234 poor households were filed in the poverty registration, 323 poor households were not filed in the poverty registration, while 503 non-poor households were filed in the poverty registration. This indicates that there are deviations in the precise identification mechanism.

Table 2 compares the family statuses of poor households and the rural households filed in the poverty registration. The following problems are revealed by the table: (1) The household resource endowment directly affects the financial conditions of rural households. The per-household and per-capita amount of arable land areas owned by the poor households were both higher than the values of the whole sample. This may have indirectly indicated that the poor households cannot acquire wealth only by farming. The per-household and per-capita value of houses owned by the poor households were both lower than that of the entire sample and the rural households filed in the poverty registration. This reflects that most of the poor households do not have many valuable fixed assets. (2) The transfer of surplus rural labor to non-agricultural industries can increase income. However, the number of people from poor households engaged in non-agricultural industries is much lower than that of the whole sample; therefore, they are unable to produce stable cash flows for their families. (3) From the perspective of income, the average income of poor households in the previous year was 6776.99 RMB, only equal to 17.62% of the entire sample and 26.17% of the rural households filed in the poverty registration. From the perspective of expenditure, the expenditure of poor households was much greater than their income at 2.71 times their income, while the expenditure-to-income ratio of the rural households filed in the poverty registration was only 89.88%. On the one hand, this shows that poor households are indeed in dire situations. On the other hand, it shows that there is a certain degree of deviation in the precise identification mechanism, and that the really poor households have not been correctly identified. (4) The proportions of plains in the areas where poor households and the rural households were filed in the poverty registration were lower than the average of the entire sample. The former is only one-half of the latter. This shows that natural resources are scarce in impoverished areas with mountainous terrains, contributing towards poverty. (5) The living conditions, assets, family health, education, and other indicators of poor households classified by income are generally lower than those of non-poor households, registered households, and non-registered households.

**Table 2:** Comparison of the statuses of poor households.

	Whole sample	Poor households	Non-poor households	Rural households filed in poverty registration	Rural households not filed in poverty registration
Arable land areas per household	4.03	4.35	3.93	3.35	4.01
Arable land areas per capita	0.94	1.01	0.92	1.03	0.94
House value per household	9.30	4.74	10.72	7.75	10.34
House value per capita	2.18	1.10	2.52	1.67	2.18
Total number of family members per household	4.26	4.30	4.25	3.97	4.38
Number of members that can work per household	2.29	2.08	2.36	2.04	2.39
Members engaged in non-agricultural industry per household	1.01	0.23	1.26	0.64	1.16
Total income per household	38469.61	6776.99	47022.32	25894.39	43388.90
Total expenditure per household	28965.63	18376.33	31823.30	23275.13	31191.69
Terrain					
Proportion of plains	10.42%	4.31%	12.06%	4.61%	12.69%
Proportion of hills	3.85%	1.62%	4.46%	0.95%	4.99%
Proportion of mountain areas	85.73%	94.08%	83.48%	94.44%	82.32%
Living conditions of farmers					
Households with clean drinking water source	87.90%	85.25%	88.61%	86.30%	88.53%
Households with access to electricity	98.85%	95.56%	98.93%	98.51%	98.99%
Households that use non-traditional fuels (such as natural gas)	28.13%	14.90%	37.08%	22.52%	36.22%
Households with a separate bathroom	60.00%	34.42%	66.90%	45.91%	65.51%
Farmers’ assets					
Houses with household appliances (such as TV, refrigerator, and washing machine)	85.81%	59.78%	92.83%	79.24%	88.38%
Family health status					
Households with all family members in a healthy state (i.e., no serious illness, long-term chronic disease, or infirmity)	52.65%	46.68%	54.26%	43.83%	56.10%
Education status of farmers					
Households where the family labor force has completed compulsory primary education	68.63%	39.21%	76.59%	63.03%	70.82%
Households with all school-age children (6–15 years old) in school	82.19%	61.89%	88.67%	79.02%	83.43%

#### Analysis of government policy implementation

Farmers must first understand targeted poverty alleviation policies before they can make decisions based on relevant information. Compared with the value of the whole sample, the rural households filed in the poverty registration system had a very high degree of understanding of the policies. Among them, the people who fully understood or had a relatively better grasp of the policies accounted for 26.62%, far exceeding the 14.60% of the whole sample. In contrast, only 11.07% of the poor households had the same levels of understanding. This shows that due to the imbalance in the supply of government public information services, a large number of poor households have a poor understanding of the government’s poverty alleviation policies, let alone the ability to apply for the poor household identification that suits them. Exactly 77.16% of the rural households filed in the poverty registration believed that the villagers in their areas can participate in the democratic appraisal of poor household identification, which was higher than the 64.90% of the entire sample and the 50.40% of the poor households. At the same time, 77.70% of rural households filed in the poverty registration believed that the list of poor households in their area would be publicized after assessment, which was higher than that of the total sample (65.53%) and than that of the poor households (61.74%). Government services in areas with registered rural households tend to be more fair and transparent, which makes it easier for poor farmers to be accurately identified.

### Econometric model

As the three dependent variables involved in this paper are all 0–1 variables, we used the Probit model to estimate the influence of government service equity on the identification of poor households. It is assumed that the standard normal probability distribution is presented for farmers identified as poor households, and Eq (1) can be obtained as follows:

$$P(T_{i}=1|Z_{i})=F(Z_{i})=\frac{1}{\sqrt{2\pi }}\int_{-\infty }^{Z_{i}}e^{-s^{2}/2}ds$$ (1)

After transforming Eq (1) using the inverse function, Eq (2) can be obtained:

$$Z_{i}=F^{-1}(P)=\alpha_{0}+\alpha_{T}I_{T}+\alpha_{T}X_{T}+u_{T}$$

where T_i_ includes the dependent variable of Y1, Y2, and Y3. Specifically, Y1 refers to “whether a non-poor household was identified as a poor household,” Y2 refers to “whether there was a failure to identify a poor household as a poor household,” and Y3 refers to “whether the identification of poor household identification was conducted wrongly.” I_T_ denotes government service equity, and X_T_ refers to the other factors affecting the identification of poor households. α_0_ is the intercept term, α_T_ represents the estimated coefficient of government service equity, and μ_T_ is the random disturbance term.

### The impact of rural households’ participation in the identification of poor households from the perspective of farmers

#### Benchmark regression

Based on the characteristics of field survey data, we used the Probit model to analyze the influencing factors of poor household identification. In Table 3, the column of Model (1) shows the results of a regression using the dummy variable of “identifying a non-poor household as a poor household”; the column of Model (2) shows the results of a regression using the dummy variable of “failure to identify a poor household as a poor household”; the column of Model (3) shows the results of a regression using the dummy variable of “one of the above two situations occurs.”

**Table 3:** Regression results.

Variable	Model (1)	Model (2)	Model (3)
Participation in appraisals	0.129***	-0.025***	0.089***
	(0.014)	(0.008)	(0.020)
Gender	0.011	0.003	0.031
	(0.018)	(0.009)	(0.023)
Age	-0.000	-0.001**	-0.002**
	(0.001)	(0.000)	(0.001)
Marital status	-0.043**	0.026***	0.007
	(0.023)	(0.007)	(0.027)
Educational level	0.002	-0.000	-0.003
	(0.010)	(0.005)	(0.012)
Understanding of policies	0.061***	-0.012***	0.043***
	(0.009)	(0.004)	(0.010)
Family size	-0.014**	0.016***	0.021***
	(0.006)	(0.003)	(0.007)
Labor size	-0.033***	0.005	-0.014
	(0.009)	(0.004)	(0.011)
Non-agricultural employment	-0.042***	-0.013**	-0.046***
	(0.011)	(0.005)	(0.014)
Land size	-0.002	0.001	-0.001
	(0.002)	(0.001)	(0.002)
Houses value	-0.019***	-0.005	-0.029***
	(0.008)	(0.003)	(0.009)
Family income	0.083***	-0.074***	-0.100***
	(0.011)	(0.008)	(0.013)
Family expenditure	-0.044***	0.008*	-0.018
	(0.010)	(0.005)	(0.013)
Plains	-0.127***	-0.010	-0.186***
	(0.015)	(0.013)	(0.025)
Hills	-0.152***	0.002	-0.227***
	(0.011)	(0.020)	(0.027)

Note:

*, **, and *** represent significance at the 10%, 5%, and 1% level, respectively. Standard deviations reported in parentheses.

It can be seen from Table 3 that the coefficients of participation in appraisals are all significant on the 1% level, regardless of whether other control variables are added. This shows that poor household identification will be significantly affected by participation during the implementation of the targeted poverty alleviation policies. The regression results of Model (1) show that participation in appraisals significantly increases the probability of identifying a non-poor household as a poor household, which verifies Hypothesis 2 of this study. The regression results of Model (2) shows that participation in appraisals significantly decreases the probability of failing to identify a poor household as a poor household, which verifies Hypothesis 3 of this study. The regression results of Model (3) show that in general, participation in appraisals increases the probability of deviations in poor household identification. This rejects Hypothesis 1 of this study. A possible reason is that people participating in the appraisals (including those from poor households and non-poor households) tend to fight for their own interests. As a result, the resources will be seized by elite farmers to a certain extent, while this is consistent with the findings of other scholars [9, 15].

According to the control variables, when the head of a household is older, the probability of the poor household being omitted from poor household registration will be lower, while the probability of deviation in poor household identification will be lower. If the head of the household is married, the probability of being identified as a poor household will be lower, both for poor households or non-poor households. The influence coefficients of the variable of “degree of understanding the policies” are 0.061, -0.012 and 0.043 respectively, which are significant at the 1% significance level. This shows that as a household knows more about the targeted poverty alleviation policies, it will have a higher probability to be identified as a poor household, while the probability of deviation in poor household identification will be higher. The regression results show that as the total number of family members increase, the probability of a rural household being identified as a poor household will decrease, but the probability of deviation in poor household identification will increase. Family members that can work provide for the family. When a family has more members that can work, it effectively decreases the probability of a non-poor household being identified as a poor household. With high population density and a limited supply of land, China faces industrial restructuring, and a large number of rural surplus labors are transferred to secondary and tertiary industries. At the same time, due to the low efficiency of agricultural production, the income from working in non-agricultural industries is more than that from farming. Therefore, as more people are engaged in non-agricultural industries, the probability of rural households being identified as poor households will decrease, and the probability of deviation in poor household identification will also decrease. The coefficients of house value in Model (1) and Model (3) are -0.019 and -0.029, which are significant at the 1% level, indicating that an increase in house value will decrease the probability of identifying the non-poor households as poor households, and will decrease the probability of deviation in poor household identification. The probability of identifying the rural households with high income as poor households may increase, but the increase in income will reduce the probability of deviation in poor household identification as a whole. The increase in expenditure will reduce the probability of rural households being identified as poor households, which is consistent with the study of Yin and Guo [1]. This shows that the natural conditions of an area will inevitably affect the economic development of the region. Non-poor households in mountainous areas are more likely to be identified as poor households than the non-poor households in plains and hills, but the probability of deviation in poor household identification in the mountainous areas is also lower.

#### Robustness test

The first three columns of Table 4, namely Model (4), Model (5), and Model (6), report the estimated results using government failure as a substitute for participation in appraisals as the core independent variable. Several coefficients of government failure are statistically significant at the level of 1% and are basically consistent with the estimated results in Table 3. Among them, the regression results of model (4) show that government failure will significantly increase the probability of not being poor but being identified as poor, which verifies hypothesis 2 of this study. The regression of model (5) shows that government failure reduces the probability of being poor but not being identified as poor, which verifies hypothesis 3 of this paper. The regression of model (6) shows that government failure increases the probability of poverty identification bias on the whole, which negates hypothesis 1 of this paper.

**Table 4:** Robustness test results.

	Replacing the core independent variable			Replacing the dependent variable		
Variable	Model (4)	Model (5)	Model (6)	Model (7)	Model (8)	Model (9)
Participation in appraisals				0.116***	-0.059***	0.064***
				(0.014)	(0.012)	(0.022)
Government failure	0.152***	-0.023***	0.149***			
	(0.014)	(0.008)	(0.019)			
Gender	-0.008	-0.001	0.016	0.011	0.000	0.009
	(0.019)	(0.008)	(0.024)	(0.018)	(0.011)	(0.026)
Age	0.000	-0.001***	-0.001	-0.001	0.000	-0.001
	(0.001)	(0.000)	(0.001)	(0.001)	(0.000)	(0.001)
Marital status	-0.051**	0.027***	0.003	-0.065***	0.002	-0.056***
	(0.023)	(0.006)	(0.027)	(0.024)	(0.012)	(0.030)
Educational level	0.002	-0.002	0.003	-0.003	0.005***	0.002
	(0.010)	(0.005)	(0.013)	(0.009)	(0.006)	(0.013)
Understanding of policies	0.051***	-0.011***	0.035***	0.046***	-0.027	0.012
	(0.008)	(0.004)	(0.010)	(0.007)	(0.005)	(0.011)
Family size	-0.017***	0.017***	0.020***	0.005	-0.004	0.004
	(0.006)	(0.003)	(0.007)	(0.006)	(0.003)	(0.008)
Labor size	-0.031***	0.005	-0.014	0.002	0.006	0.000
	(0.009)	(0.004)	(0.011)	(0.009)	(0.005)	(0.012)
Non-agricultural Employment	-0.043***	-0.012**	-0.045***	-0.052***	0.007	-0.039***
	(0.011)	(0.005)	(0.014)	(0.010)	(0.006)	(0.014)
Land size	-0.002	0.001	-0.001	-0.001	-0.002	-0.002
	(0.002)	(0.001)	(0.002)	(0.001)	(0.001)	(0.003)
House value	-0.012*	-0.006*	-0.024***	0.079***	-0.114***	-0.125***
	(0.007)	(0.003)	(0.009)	(0.008)	(0.008)	(0.010)
Family income	0.098***	-0.075***	-0.090***	-0.033***	0.008	-0.013
	(0.011)	(0.008)	(0.012)	(0.009)	(0.005)	(0.013)
Family expenditure	-0.042***	0.007	-0.016	-0.047***	0.012**	-0.031**
	(0.010)	(0.004)	(0.012)	(0.009)	(0.006)	(0.013)
Plains	-0.139***	-0.006	-0.194***	-0.056**	-0.045***	-0.223***
	(0.012)	(0.014)	(0.024)	(0.022)	(0.009)	(0.026)
Hills	-0.150***	-0.005	-0.228***	-0.144***	0.211***	0.167***
	(0.010)	(0.018)	(0.026)	(0.013)	(0.057)	(0.057)

Note:

*, **, and *** represent significance at the 10%, 5%, and 1% level, respectively. Standard deviations are reported in parentheses.

The last three columns of Table 4, namely, Models (7), (8), and (9), report the estimated results of using assets as an alternative to per capita income for the classification of poor and non-poor households. In reality, housing is the asset owned by the highest proportion of Chinese residents, so it is appropriate to select house values for asset classification. We classified those with house values below 25% as poor (682 samples) and the rest as non-poor (1,939 samples). From the regression results, regardless of whether the classification criteria of poor households were changed or not, participation in appraisals was statistically significant at the level of 1%, which was consistent with the baseline regression in Table 3.

#### Propensity score matching (PSM)

**Fig 2:**
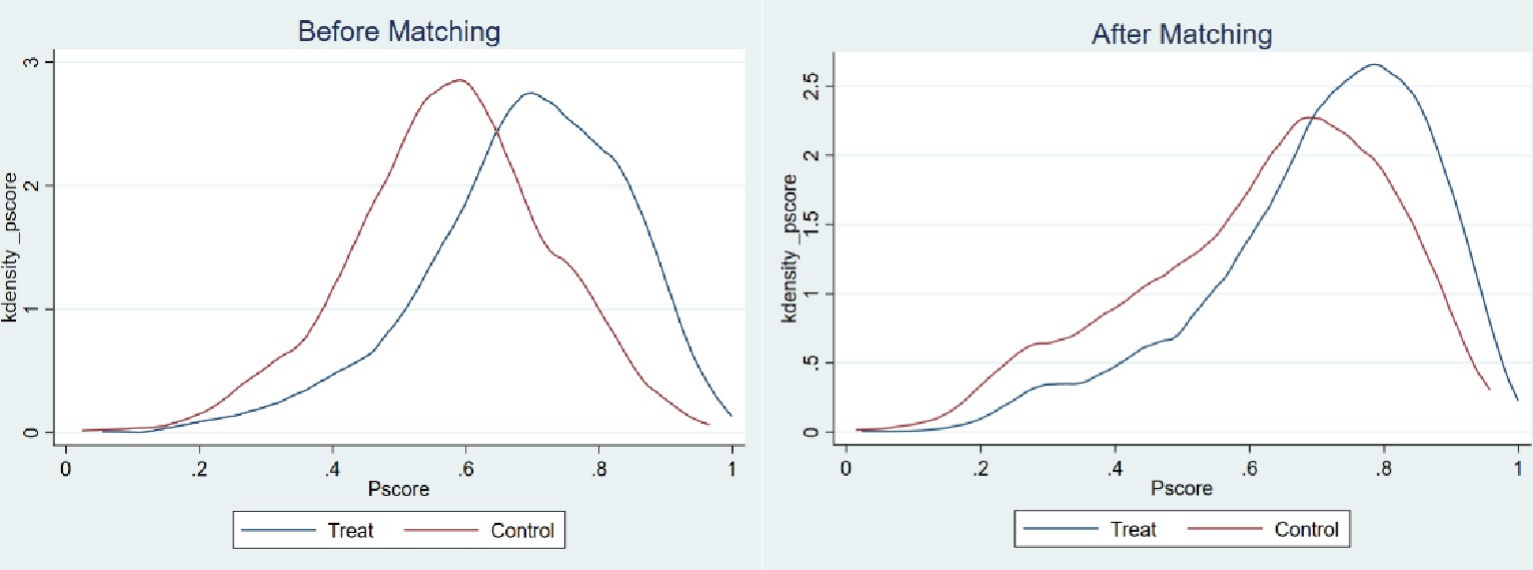
Y1: Comparison of the PS probability values between “rural households who participated in the appraisals” and “rural households who did not participate in the appraisals” before and after nearest neighbor matching.

**Fig 3:**
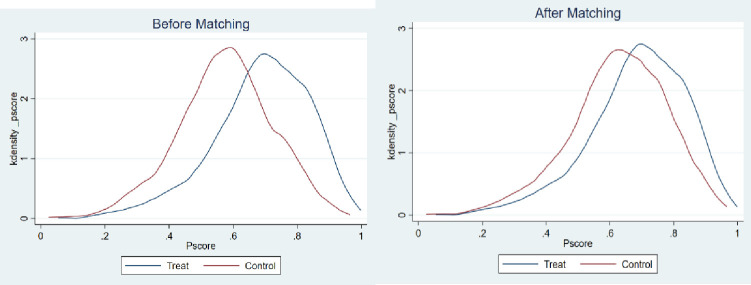
Y2: Comparison of the PS probability values between “rural households who participated in the appraisals” and “rural households who did not participate in the appraisals” before and after nearest neighbor matching.

**Fig 4:**
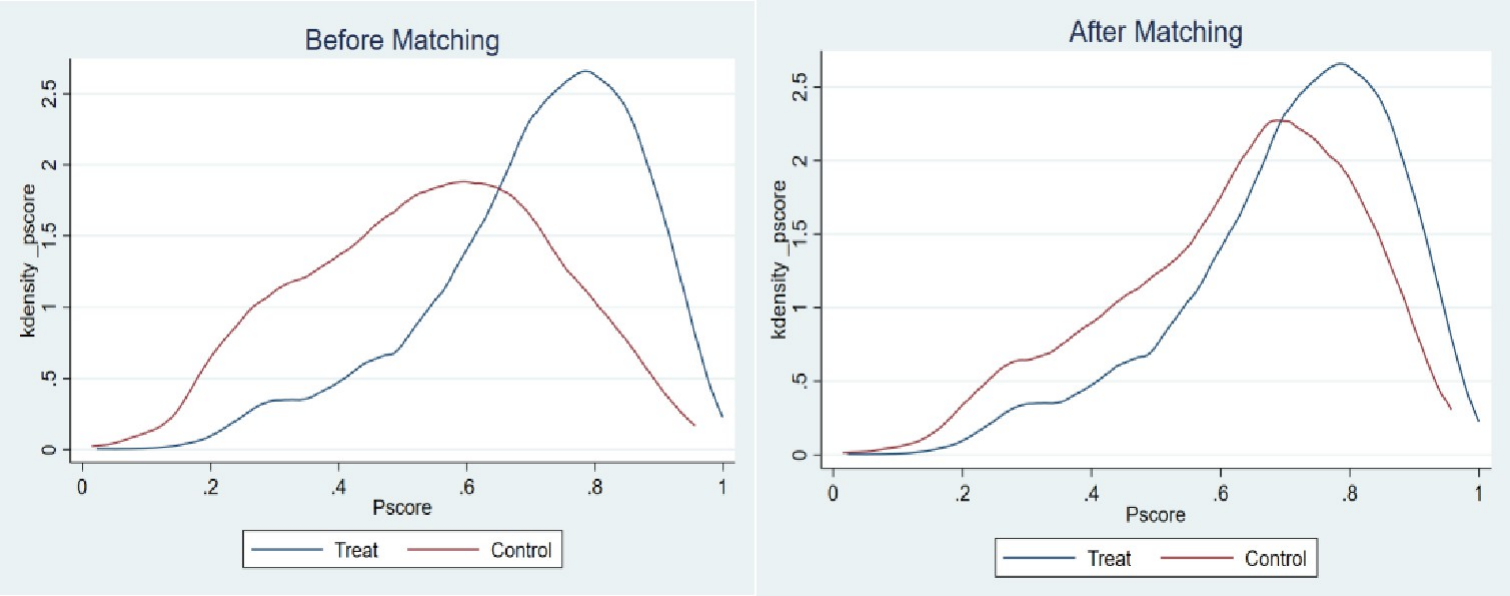
Y3: Comparison of the PS probability values between “rural households who participated in the appraisals” and “rural households who did not participate in the appraisals” before and after nearest neighbor matching.

In order to deal with the potential endogeneity of the variables and self-selection problems in regression estimations, PSM is adopted to verify the impact of massive participation in appraisals on the farmers’ probabilities of being identified as poor households. First of all, the research objects were divided into two groups: the rural households who participated in the appraisals and the rural households who did not participate in the appraisals. The treatment condition in this study was defined according to the nature of dichotomous variables. The rural households who participated in the appraisal belong to the treatment group, while the rural households who did not participate in the appraisals belong to the control group. To evaluate the average treatment effect on the treated (ATT), multiple covariates should be controlled to match up to the members from the two different groups, that is, the rural households who participated in the appraisals and the rural households who did not participate in the appraisals, while the matching criterion have the most similar characteristics. According to the aforementioned Probit model regression, the selected covariates include all control variables. Secondly, taking the nearest neighbor matching as the example, kernel density plots before and after the propensity score matching were drawn for the three cases of poverty identification according to the setting of dependent variables. Figs 2–4 represent the kernel density plots in the case of Y1, Y2, and Y3, respectively. And the left image in Figs 2–4 shows the kernel densities of PS of the treatment group and the control group before matching, while the right image shows the comparison of the kernel density functions after matching. It can be found that before matching, no matter which group of graphs, the differences in sample characteristics of the treatment group and the control group are relatively larger. If estimations are done directly, biased statistical inference may be obtained. In contrast, the sample characteristics of the two groups are closer after matching, and the matching effect is better.

Finally, after the above operations, the nearest neighbor matching method (without replacement), the radius matching method (r = 0.05), and kernel matching method were formally used for estimation. As shown in Table 5, participation in the appraisals has a significant effect on increasing the probability of being identified as a poor household. After the propensity score matching, the average treatment effects on the (ATT) treatment group reached about 14%, 5%, and 10%, respectively. In other words, if a non-poor household participates in the appraisals, the probability of being identified as a poor household will increase by 14%. If a poor household does not participate in the appraisals, the probability of being identified as a poor household will decrease by 5%. Participation in the appraisals will increase the occurrence of one of the above two situations by 10%. In contrast, the Probit model regression produced certain underestimated results.

**Table 5:** Propensity score matching estimation results.

Dependent variable		Treatment effect	Treatment group	Control group	Difference	Standard error	T test value
Nearest neighbor matching	Y1	Before matching	0.251	0.082	0.169	0.016	10.71
		ATT	0.251	0.109	0.142	0.018	7.99
Radius matching		Before matching	0.251	0.082	0.169	0.016	10.71
		ATT	0.251	0.111	0.141	0.017	8.43
Kernel matching		Before matching	0.251	0.082	0.169	0.016	10.71
		ATT	0.251	0.113	0.139	0.017	8.27
Nearest neighbor matching	Y2	Before matching	0.080	0.203	-0.123	0.013	-9.28
		ATT	0.080	0.134	-0.054	0.021	-2.64
Radius matching		Before matching	0.080	0.203	-0.123	0.013	-9.28
		ATT	0.080	0.132	-0.052	0.019	-2.70
Kernel matching		Before matching	0.080	0.203	-0.123	0.013	-9.28
		ATT	0.080	0.133	-0.053	0.019	-2.73
Nearest neighbor matching	Y3	Before matching	0.331	0.285	0.046	0.019	2.43
		ATT	0.331	0.232	0.100	0.026	3.88
Radius matching		Before matching	0.331	0.285	0.046	0.019	2.43
		ATT	0.332	0.235	0.097	0.024	4.00
Kernel matching		Before matching	0.331	0.285	0.046	0.019	2.43
		ATT	0.332	0.238	0.094	0.024	3.87

### Impact of the governments’ targeted poverty alleviation policies on poor identification of poor households from the perspective of grassroots officials

With the existence of the aforementioned government failures, what is the implementation effect of the targeted poverty alleviation policies? Have the policies really alleviated poverty in an effective way? To answer the questions, this section further analyzes the implementation effect of the government’s poverty alleviation policies. On the whole, officials gave low evaluations to the implementation of the diversified and targeted poverty alleviation policies. Only two people believed that the current policies of the government were remarkable, while 14 people believed that the policies were fine; 181 people believed that the policies were extremely bad, while 311 people believed that they were relatively bad, and the two groups together accounted for 71.51% of all respondents. This is a shocking result. Why is the evaluation so low despite a great deal of effort devoted by governments at all levels in China? Which part of the work went wrong? This is worthy of in-depth study and discussion. Table 6 lists the grassroots officials’ evaluations of various targeted poverty alleviation policies of the governments. From the perspective of the respondents, the low efficiency and lack of supervision of China’s grassroots-level governments have made it impossible to guarantee the correct allocation and use of poverty alleviation funds or to ensure the effective implementation of poverty alleviation policies.

**Table 6:** Grassroots officials’ evaluations of the governments’ targeted poverty alleviation policies.

	Remarkable	Fine	Ordinary	Relatively bad	Extremely bad
Improve infrastructure	0.58%	4.36%	30.23%	41.57%	23.26%
Poverty alleviation by the development of production	1.16%	9.59%	30.23%	38.37%	20.93%
Poverty alleviation by relocation	2.47%	10.47%	31.98%	34.59%	20.49%
Poverty alleviation by ecological compensation	3.20%	13.23%	36.34%	30.23%	17.01%
Poverty alleviation by education development	0.58%	7.12%	44.04%	42.73%	20.06%
Social security	0.44%	2.47%	29.22%	40.12%	27.76%
Poverty alleviation with transfer of labor	2.18%	13.23%	37.21%	31.10%	16.28%
Poverty alleviation with financial measures	5.09%	9.01%	36.19%	32.12%	17.59%
Government supervision	0.44%	2.47%	26.60%	39.97%	30.52%
Overall evaluation	0.29%	2.03%	26.16%	45.20%	26.31%

Nearly one-third of the respondents believed that the diversified and targeted poverty alleviation policies of the governments in their regions were simplified. For example, the policies were simplified into providing funds and means of production or daily necessities, instead of being tailored to meet the actual needs of individual rural households and achieve targeted poverty alleviation. Correspondingly, as many as 60% of the grassroots officials highlighted that understanding the actual needs of poor households should be given priority in the implementation of targeted poverty alleviation policies before considering the amount of available poverty alleviation funds and the degree of difficulty in implementing the policies, while the potential benefits for the government should be the last thing to aim for. This shows that the grassroots-level governments in poverty-stricken areas are still obsessed with numbers. They care more about political performance and the degree of difficulty in implementing the policies than the true situations of the poor households, so that the diversified and targeted poverty alleviation policies have become empty shells and go against the original intention of targeted poverty alleviation proposed by the central government. Even so, most of the respondents said that the implementation of the government’s current diversified and targeted poverty alleviation policies had alleviated the poverty of the local communities. However, nearly one-fifth of the people had opposite opinions. The main reasons were that the targeted poverty alleviation policies could not satisfy the actual needs of poor households, the poverty alleviation resources were misappropriated by government departments and elite farmers instead of being actually allocated to the households, while the officials stationed in the villages had little enthusiasm for the work. Other reasons included the difficulties in implementing the policies, the lack of a sustainable and effective income-increasing mechanism, as well as procedural works. Exactly 39.53% of the grassroots officials pointed out that the local governments and relevant departments failed to publicize information on poverty alleviation funds, materials, and projects regularly. Exactly 16.86% believed that there was corruption in the implementation of targeted poverty alleviation policies in their regions. The grassroots-level governments are the public faces of the party and the state to the people. Any possible corruption in the implementation of poverty alleviation policies brings about pernicious effects to the credibility of the party and the state.

### Further discussion: The significance of accurate identification for preventing rural households returning to poverty after escaping it

**Fig 5:**
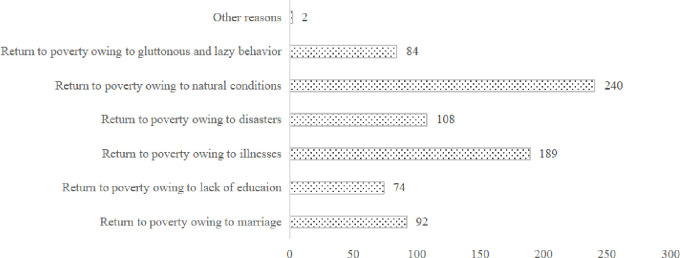
Distribution of reasons for rural households returning to poverty after escaping it in the sample.

The current targeted poverty alleviation policy is a dynamic mechanism for poor households to be identified and assisted. Although China has achieved full poverty alleviation in 2020, according to the survey, many rural households will not continue to receive the previous level of policy assistance after escaping poverty. Due to the lack of sustainable assistance policies, the assisted farmers are prone to falling back into poverty because they do not have the ability to resist risks [46]. In our survey, 317 grassroots officials said that there were many poor families in their areas who had been lifted out of poverty and then returned to poverty. As shown in Fig 5, this is usually caused by the inability of natural conditions to develop, or by diseases or disasters among poor farmers. Given this, an accurate identification mechanism should also be established for those rural households returning to poverty, so that the government can implement effective assistance. Therefore, the analysis results of this paper are of great significance to China’s social development in the post-poverty alleviation period.

## Conclusions and policy implications

### Conclusions

Based on a total of 688 samples of grassroots officials, as well as 2,621 samples of rural households, this study used the Probit model to analyze poor household identification and the influencing factors of the precise identification mechanism in the hope of identifying the core variables. In this way we hoped to provide empirical references for optimizing the targeted poverty alleviation mechanism. The conclusions of this study are as follows:

1. From the different perspective of grassroots officials and rural households, it was found that during the implementation of the targeted poverty alleviation policies, identification of poor households is significantly impacted by the participation of rural households in the appraisal. As the supply of public information by the government is insufficient, while some grassroots officials fail to maintain fairness and openness when implementing policies, poor households as information vulnerable groups have poor understanding of the targeted poverty alleviation policies, and may not be able to participate in the poor household identification process. As a result, the identification results are quite different from the real situation, which is problematic if the government aims for poverty alleviation measures to be effective and equitable.
2. The empirical results show that participation in appraisals significantly increases the probability of identifying a non-poor household as a poor household; participation in appraisals significantly decreases the probability of failing to identify a poor household as a poor household; and in general, participation in appraisals increases the probability of deviations in poor household identification. The above results are still valid after dealing with the self-selection problem. In addition, factors such as family statuses and natural conditions also affect poor household identification.

### Policy implications

According to the above conclusions, the policy implications of this paper are as follows.

1. Strengthen the publicity and guidance to improve the farmers’ knowledge of poverty alleviation policies, and measures should be taken to enable farmers to effectively participate in the identification process of poor farmers. The policymakers have good intentions when formulating policies, while the details of implementation are usually idealized. However, due to the existence of information asymmetry and adverse selection problems in economics, the failure of the allocation of poverty alleviation resources will usually happen eventually. Therefore, when developing and implementing precise identification mechanisms, governments of developing countries, including that of China, should start by ensuring the wide publication of the policies using multiple channels.
2. Focus on nurture of officials and build a mighty army for the battle of poverty alleviation. In developing countries, grassroots officials are the representatives of the government for the people. If grassroots officials are unprofessional and have low political awareness and poor understanding of poverty alleviation policies, the state’s poverty alleviation policies will easily become empty talk. The formulation of a policy is undoubtedly important, while the means of implementation and the people responsible for the implementation are also crucial [16].
3. Perfect the top-level design and promote diversified and targeted poverty alleviation policies. China and many developing countries have vast territories, and the situations in provinces and cities are different. Regional governments should formulate well-targeted and diversified poverty alleviation policies according to their own natural resource endowments and reasons of poverty [40].
4. Precision is at the core of developing countries’ targeted poverty alleviation policies. Precise identification, assistance, management, and assessment should be achieved in the course of poverty alleviation development. Therefore, it is necessary to construct a dynamic management mechanism for the “entry-exit” of poor households with the use of scientific measurement and big data analysis [18, 23]. When implementing poverty alleviation policies in developing countries, it is necessary to tailor measures according to individual needs to resolve the difficulties faced by poor households in reality.
5. In order to prevent rural households from returning to poverty for various reasons after having previously escaped it, the government should continue to provide some necessary assistance in industrial support, vocational skills training, social security, job creation, and relocation subsidies after they are lifted out of poverty, so as to reduce the risk of poverty-stricken rural households returning to poverty again.
